# Unilateral transverse testicular ectopia with recurrence of inguinal hernia: a case report

**DOI:** 10.1186/s13256-023-03782-8

**Published:** 2023-02-27

**Authors:** Mahsa Gholizadeh, Ali Mohammad Fakhre yasseri

**Affiliations:** 1 Alumna of Medicine, Tehran University of Medical Sciences, Tehran, Iran; 2 Shariati Hospital, Alborz University of Medical Sciences, Karaj, Alborz Province Iran

**Keywords:** Testis, Undescended testis, Transverse testicular ectopia, Surgical technique, Case report

## Abstract

**Background:**

Crossed testicular ectopia or transverse testicular ectopia is an extremely rare urogenital anomaly. In this condition, on average at 4 years of age the testes migrate through the inguinal canal and one or both testes may turn up in the abdomen, inguinal region, or in the hemiscrotum, with an empty contralateral hemiscrotum. Our case report documents transverse testicular ectopia in a 5-year-old boy who presented with right inguinal hernia and nonpalpable left testis. He underwent previous right herniorrhaphy at the age of 1 year.

**Case presentation:**

A 5-year-old Iranian boy was diagnosed with a right inguinal hernia. He underwent right inguinal herniorrhaphy at the age of 1 year. For this case report, the hernia symptoms had returned. Both testicles were palpated in the right scrotum, an ultrasound examination also revealed both testicles to be present in the right scrotum, and a hernia sac located in the right inguinal region with an internal ring. The patient was recommended to undergo a surgical reconstruction. Surgical reconstruction was performed by crossing the left testis in the transseptal orchiopexy technique.

**Conclusion:**

In patients with cryptorchidism on one side and an inguinal hernia on the other side, the surgeon must consider a rare condition known as transverse testicular ectopia. Sonography can be helpful for diagnosing cases where transverse testicular ectopia is suspected, evaluating other anomalies, and selecting the most appropriate treatment.

## Introduction

Transverse testicular ectopia (TTE), or crossed testicular ectopia, is an extremely rare urogenital anomaly; the first report of this condition was in 1886 by Von Lenhossek. The other terms used to describe it in the literature are unilateral double testis, testicular pseudo-duplication, and transverse aberrant testicular maldescent [1–3]. The mean age at which the abnormality manifests is 4 years, and it manifests in several ways, including one or both testicles misplaced in the abdomen or inguinal area, or the descent to the hemiscrotum with an empty contralateral hemiscrotum [4, 5]. Most often, this disease is diagnosed during surgery for a hernia or undescended testis [6, 7]. In this case study, we report a case of type one transversal testicular ectopia in a 5-year-old boy who underwent a previous right herniorrhaphy at the age of 1 year.

## Case presentation

A 5-year-old Iranian boy was diagnosed with a right inguinal hernia. Previously, at the age of 1 year, he underwent right inguinal herniorrhaphy. The child’s parents mention that, after the surgery at the age of 1 year, swelling occasionally occurs in the right inguinal region. As part of the examination, both testicles were palpated in the right scrotum. Throughout the examination, all other aspects were normal. All laboratory test results were normal, and the child’s parents do not mention a history of TTE in the family.

Ultrasound showed that two testicles are located in the right scrotum and the hernia sac is located in the right inguinal area, with an internal ring diameter of 9 cm. Two spermatic cords were also observed on the right inguinal canal.

**Fig. 1:**
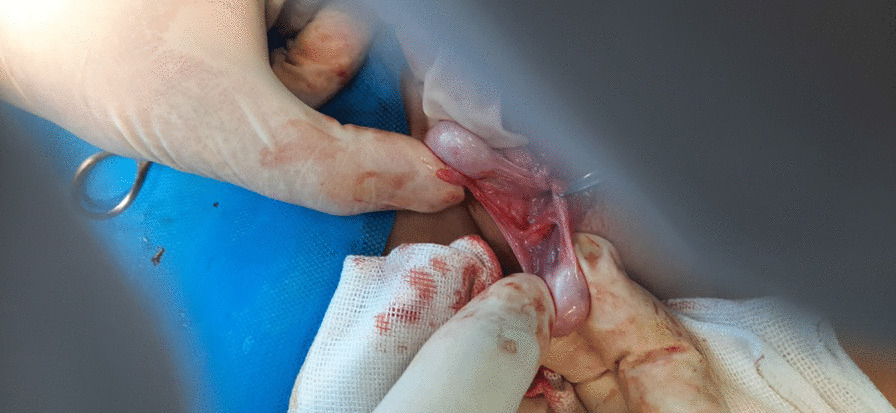
Delivery of two testicles

**Fig. 2:**
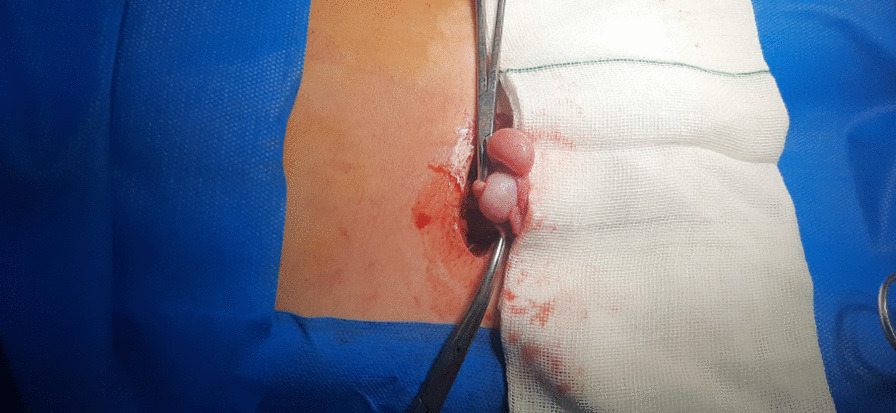
Release of two spermatic cords

**Fig. 3:**
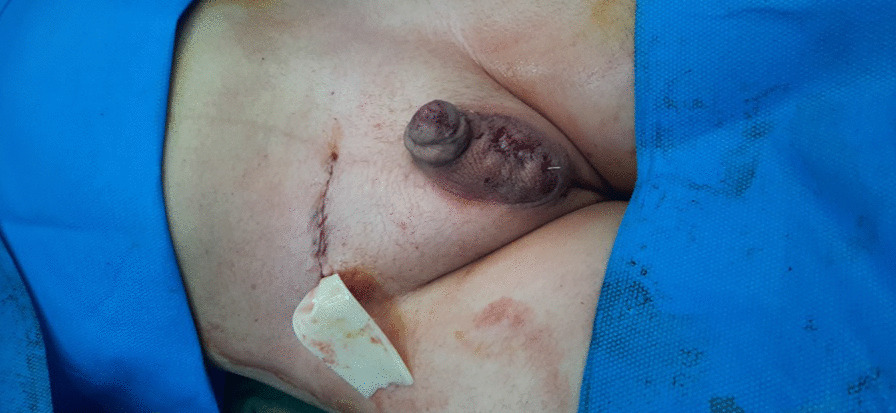
Postoperative view

**Fig. 4:**
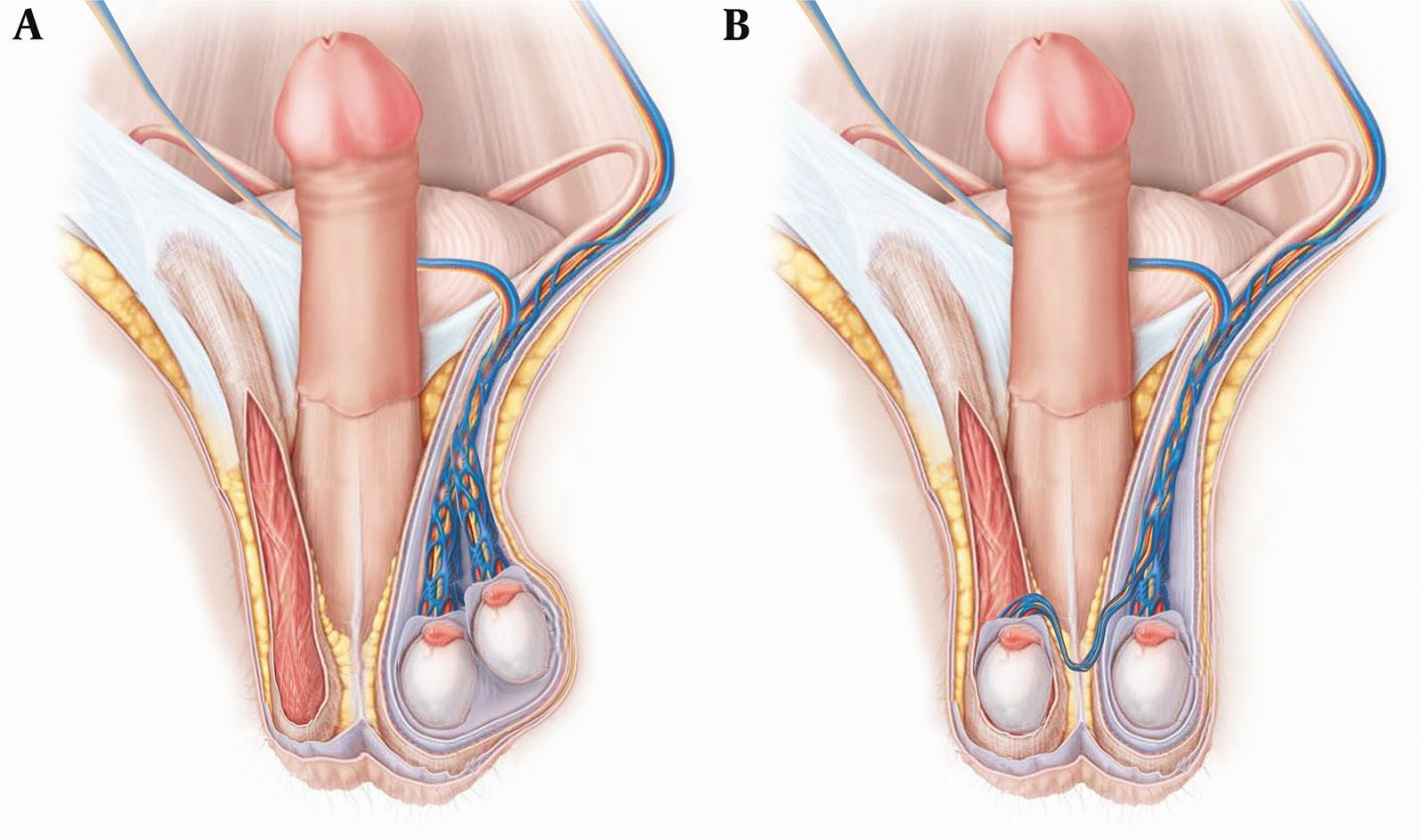
Schematic drawing of transverse testicular ectopia. **A** Preoperative. **B** Postoperative

The patient was recommended to undergo surgical reconstruction. We chose the type 1 technique (transseptal orchiopexy) for surgery. A right inguinal incision was made at the site of the previous scar. It was evident that the previous surgery had caused adhesions. The layers were opened according to anatomic construction. The hernia sac was opened from the scar site of the previous operation, and the spermatic cords were completely separated from it. During the release of the spermatic cords, care was taken to prevent damage to the cords. The testicles were delivered and dissected (Figs. 1, 2). Because two testicles were located in the right scrotum, it was necessary to make a mid-raphe incision. Through a hole in the middle of the raphe, one of the testicles was transferred to the left hemiscrotum, and fixed with Prolene suture. Also, the right hemiscrotum was fixed with the right testicle. We fixed a Penrose drain for removing blood and other fluids out of the surgical area, to prevent infection. For the closure of the fascia, subcutaneous tissue, and skin, a sterile bandage was applied (Fig. 3). As soon as the drain was removed, the patient was discharged. One week later, the patient’s testicles were in their proper hemiscrotums, and no complications were observed (Fig. 4). Follow-up with the patient occurs at 1 year intervals to check for possible complications. The case is briefly described in Table 1.

**Table 1:** Case summary

	1 year old (*T*_0_)	5 years old (*T*_1_)
Symptoms	Swelling in the right inguinal area	Swelling in the right inguinal area
Examination	Swelling in the right inguinal area (no further information is provided)	Both testicles were palpated in the right scrotum, left scrotum was empty
Sonography	There is no information in the previous document	Two testicles were located in the right scrotum, the hernia sac was located in the right inguinal area, with an internal ring diameter of 9 cm, left scrotum was empty
Surgery	Only right herniorrhaphy	Transseptal orchiopexy (Ombredanne’s technique)
Complication	Right hernia return	After surgery, no complications were observed, follow-up with the patient at 1 year intervals to check for possible complications

## Discussion

In the current report, we have demonstrated a case of TTE with previous herniorrhaphy. The incidence of TTE is one in 4 million in children [8]. Approximately 260 cases of transverse testicular ectopia have been reported in the literature. TTE is an extremely rare anomaly in children. The etiology of TTE has been explained by several theories. Berg *et al*. assumed that the two testes developed from the same germinal ridge [9, 10]. Thevathasan *et al.* proposed both testes, prior to descent, originated from the same vaginalis [11]. According to Kimura *et al.* [12], the vas deferens can originate unilaterally or bilaterally, but the testicle may be crossed if both originate from one side. As Gupta *et al.* point out, the Wolffian ducts become connected early in development, and as a result, when one testicle moves, the opposite testicle also moves [13].

There is always an inguinal hernia on one side because the two testicles descend through the same inguinal canal. According to Gauderer *et al.*, crossed testicular ectopia has been classified according to a variety of associated abnormalities. Type 1 has only inguinal hernias (40–50%), type 2 has Müllerian duct structures (30%), and type 3 has other genitourinary abnormalities without Müllerian remnants (20%) (hypospadias, pseudohermaphroditism, scrotal abnormalities). In line with that classification, this case was classified as type 1, which is the most common form of TTE (40–50%). TTE primarily manifests as an inguinal hernia in one side, and cryptorchidism in the other side or sometimes in both sides [14].

TTE is treated surgically using two techniques: transseptal orchiopexy and transperitoneal orchiopexy. The transperitoneal orchiopexy technique involves moving an ectopic testicle opposite the scrotal septum through a hole in the scrotal septum, while transperitoneal orchiopexy involves transferring an ectopic testis across the base of the penis into the extraperitoneal area, and fixing it to the opposite side of the scrotum [15]. A long testicular vessel and vas deferens is required to perform a transperitoneal orchiopexy procedure.

Transseptal orchiopexy (Ombredanne’s technique) is the preferred surgical technique for treating TTE. As part of the treatment algorithm for this technique, care must be taken to maintain blood supply to the vas deferens and testicles [16, 17]. Bascuna *et al.* [18] developed the algorithm for this technique, which was modified by Raj *et al.* [19]. A transseptal orchiopexy is used in this case.

Our case involves a patient who had previously undergone right herniorrhaphy in another treatment center, without attention to the importance of a complete physical examination of the patient, and without having TTE disease in mind. Examination had shown the existence of a right hernia with undescended testis on the opposite side, and this finding was confirmed by ultrasound. While complete physical examinations alone may be sufficient to make a diagnosis, the use of diagnostic tools such as ultrasound and magnetic resonance imaging (MRI) are helpful [20, 21]; however, most diagnoses occur during surgery [22].

The incidence of malignancy in patients with TTE is 18% higher than that of undescended testicular cancer [23]. Patients with TTE are also more likely to develop embryonal carcinomas, seminomas, yolk sac tumors, and teratomas [24]. Wood and Elder have demonstrated that orchiopexy performed before age 10–12 is associated with a reduced risk of undescended testicular cancer [25].

As a result of the abnormal position of the testes, these patients are at risk for fertility problems and increased risk of developing testicular cancer, so they should be followed for a period of time [2].

## Conclusion

In patients with cryptorchidism on one side, and an inguinal hernia on the other side, the surgeon must consider a rare condition known as TTE. Sonography can be helpful for diagnosing suspected cases, evaluating other anomalies, and selecting the most appropriate treatment.

## Data Availability

Not applicable.

## Abbreviations

TTE: Transverse testicular ectopia
US: Ultrasound
MRI: Magnetic resonance imaging
UDT: Undescended testicle

## References

[1] Dean GE, Shah SK (2002). Laparoscopically assisted correction of transverse testicular ectopia. J Urol.

[2] Moslemi MK, Ebadzadeh MR, Al-Mousawi S. Transverse testicular ectopia, a case report and review of literature. GMS German Med Sci. 2011;9. 10.3205/000138 PMC3141845 21808600

[3] Abdelmalak M, Waheeb S, Koraitim A, Mahdy D, Abd ElMigeid DM (2018). Two cases of transverse testicular ectopia in consanguineous boys. Eur J Pediatr Surg Rep.

[4] Barrack S (1994). Crossed testicular ectopia with fused bilateral duplication of the vasa deferential: an unusual finding in cryptochidism. East Afr Med J.

[5] Gauderer MW, Grisoni ER, Stellato TA, Ponsky JL, Izant RJ (1982). Transverse testicular ectopia. J Pediatr Surg.

[6] Jouini R, Lefi M, Sami C, Manef G, Mohsen B, Nouri A (2002). Ectopie testiculaire transverse. Prog Urol.

[7] Khalik K, Rashid R, Malik ZI (1995). Transverse ectopia of the testis: a case report. J Park Med Assoc.

[8] Hakimi T, Nijrabi M, Khalid M, Hassani G-S (2021). Transverse testicular ectopia with fused vas deferens. J Pediatr Surg Case Rep.

[9] Berg AAVIII (1904). Transverse ectopy of the testis. Ann Surg.

[10] Rajesh A, Farooq M (2017). A rare case of male pseudohermaphroditism-persistent Mullerian duct syndrome with transverse testicular ectopia—Case report and review of literature. Int J Surg Case Rep.

[11] Thevathasan C (1967). Transverse ectopia of the testis. Aust N Z J Surg.

[12] McLeod RS, Geerts WH, Sniderman KW, Greenwood C, Gregoire RC, Taylor BM (2001). Subcutaneous heparin versus low-molecular-weight heparin as thromboprophylaxis in patients undergoing colorectal surgery: results of the Canadian colorectal DVT prophylaxis trial: a randomized, double-blind trial. Ann Surg.

[13] Gupta R (1960). Ectopia testis transversa. J Ind Med Assoc.

[14] Kerigh BF, Rezaei MM (2005). Crossed testicular ectopia: a case report. Urol J.

[15] Bothra J, Shah H, Jayaswal S, Sandlas G (2014). Transverse testicular ectopia: a rare anomaly. J Pediatr Neonatal Care.

[16] Naouar S, Maazoun K, Sahnoun L, Jouini R, Ksia A, Elezzi O (2008). Transverse testicular ectopia: a three-case report and review of the literature. Urology.

[17] Sadeeq KJ, Yaqo RT, Mohammed AA (2019). Inflammatory pseudotumor of the tunica albuginea and the tunica vaginalis: case-report. Urol Case Rep..

[18] Bascuna RJ, Ha JY, Lee YS, Lee HY, Im YJ, Han SW (2015). Transverse testis ectopia: diagnostic and management algorithm. Int J Urol.

[19] Raj V, Redkar R, Krishna S, Tewari S (2017). Rare case of transverse testicular ectopia—case report and review of literature. Int J Surg Case Rep.

[20] Nam YS, Baik HK, Kim SJ, Lee HK, Park HK (1998). Transverse testicular ectopia found by preoperative ultrasonography. J Korean Med Sci.

[21] Cheikh Diop M, Ramatoulaye Ly M, Sylla Cheikhna M, Kouka SC. Bilateral pubo-penile ectopic testis: a case report. 2020.

[22] Açikalin MF, Paşaoğlu Ö, Tokar B, İlgici D, İlhan H (2004). Persistent Müllerian duct syndrome with transverse testicular ectopia: a case report with literature review. Turk J Med Sci..

[23] Berkmen F (1997). Persistent Müllerian duct syndrome with or without transverse testicular ectopia and testis tumours. Br J Urol.

[24] Eastham JA, McEvoy K, Sullivan R, Chandrasoma P (1992). A case of simultaneous bilateral nonseminomatous testicular tumors in persistent Müllerian duct syndrome. J Urol.

[25] Wood HM, Elder JS (2009). Cryptorchidism and testicular cancer: separating fact from fiction. J Urol.

